# A novel cardiovascular risk stratification model incorporating ECG and heart rate variability for patients presenting to the emergency department with chest pain

**DOI:** 10.1186/s13054-016-1367-5

**Published:** 2016-06-11

**Authors:** Micah Liam Arthur Heldeweg, Nan Liu, Zhi Xiong Koh, Stephanie Fook-Chong, Weng Kit Lye, Mark Harms, Marcus Eng Hock Ong

**Affiliations:** University of Groningen, Groningen, Netherlands; Department of Emergency Medicine, Singapore General Hospital, Outram Road, Singapore, 169608 Singapore; Centre for Quantitative Medicine, Duke-NUS Medical School, Singapore, Singapore; Department of Clinical Research, Singapore General Hospital, Singapore, Singapore; Department of Internal Medicine, University Medical Centre Groningen, Groningen, Netherlands; Health Services and Systems Research, Duke-NUS Medical School, Singapore, Singapore

**Keywords:** Heart rate variability, Emergency department, Chest pain, Risk stratification, 12-Lead ECG

## Abstract

**Background:**

Risk stratification models can be employed at the emergency department (ED) to evaluate patient prognosis and guide choice of treatment. We derived and validated a new cardiovascular risk stratification model comprising vital signs, heart rate variability (HRV) parameters, and demographic and electrocardiogram (ECG) variables.

**Methods:**

We conducted a single-center, observational cohort study of patients presenting to the ED with chest pain. All patients above 21 years of age and in sinus rhythm were eligible. ECGs were collected and evaluated for 12-lead ECG abnormalities. Routine monitoring ECG data were processed to obtain HRV parameters. Vital signs and demographic data were obtained from electronic medical records. Thirty-day major adverse cardiac events (MACE) were the primary endpoint, including death, acute myocardial infarction, and revascularization. Candidate variables were identified using univariate analysis; the model for the final risk score was derived by multivariable logistic regression. We compared the performance of the new model with that of the thrombolysis in myocardial infarct (TIMI) score using receiver operating characteristic (ROC) analysis.

**Results:**

In total, 763 patients were included in this study; 254 (33 %) met the primary endpoint, the mean age was 60 (σ = 13) years, and the majority was male (65 %). Nineteen candidate predictors were entered into the multivariable model for backward variable elimination. The final model contained 10 clinical variables, including age, gender, heart rate, three HRV parameters (average R-to-R interval (RR), triangular interpolation of normal-to-normal (NN) intervals, and high-frequency power), and four 12-lead ECG variables (ST elevation, ST depression, Q wave, and QT prolongation). Our proposed model outperformed the TIMI score for prediction of MACE (area under the ROC curve 0.780 versus 0.653). At the cutoff score of 9 (range 0–37), our model had sensitivity of 0.709 (95 % CI 0.653, 0.765), specificity of 0.674 (95 % CI 0.633, 0.715), positive predictive value of 0.520 (95 % CI 0.468, 0.573), and negative predictive value of 0.823 (95 % CI 0.786, 0.859).

**Conclusions:**

A non-invasive and objective ECG- and HRV-based risk stratification tool performed well against the TIMI score, but future research warrants use of an external validation cohort.

## Background

Chest pain is the most frequent complaint in patients over 45 years of age presenting to the emergency department (ED) in the USA [1]. The first priority in the evaluation of patients with chest pain is risk stratification to differentiate those who are in acute cardiovascular distress from those who are not. Risk stratification allows for an appropriate therapeutic strategy and an effective allocation of ED resources.

The American College of Cardiology/American Heart Association (ACC/AHA) practice guidelines recommend the use of validated risk scoring models such as the thrombolysis in myocardial infarction (TIMI), platelet glycoprotein IIb/IIIa in unstable angina: receptor suppression using integrilin (PURSUIT), and global registry of acute coronary events (GRACE) for cardiovascular risk stratification [2–6]. However, commonly employed cardiovascular risk models rely on traditional clinical indicators that are subjective, susceptible to risk factor modification (e.g., anticholesterol or antihypertensive therapy), may not be immediately available, and often do not correlate well with long-term or short-term outcomes [7–10]. Consequently, there is a need for a quantitative and rapid method to guide patient disposition in the ED.

Heart rate variability (HRV) parameters are quantitative measures of the interval between adjacent QRS complexes, mathematically derived from the electrocardiogram (ECG) [11–13]. Beat-to-beat interval fluctuations principally represent the short-term cardiovascular control exercised by the autonomic nervous system [14]. There is an increasing recognition of HRV parameters as powerful independent predictors of many cardiovascular pathological conditions [15–24], and their potential role in early cardiovascular risk stratification [25, 26].

This study aims to develop a novel risk stratification model composed of HRV parameters, demographic data, traditional vital signs and 12-lead ECG variables for the prediction of major adverse cardiac events (MACE) in patients with chest pain (henceforth referred to as the Singapore General Hospital Emergency Department risk stratification model (SEDRSM)). This study hypothesizes that the SEDRSM will perform better than an established risk stratification tool (TIMI) at predicting MACE within 30 days of a patient presenting to the ED with chest pain.

## Methods

### Design and setting

We conducted a prospective, non-randomized, observational study of patients presenting to the ED with chest pain from March 2010 until August 2015. This study was performed at the ED of the Singapore General Hospital (SGH), a tertiary care hospital in Singapore. ED triage is performed by nurses using the national Singaporean patient acuity category scale (PACS), a symptom-based triage system without strict physiological criteria. ED patients are classified with a PACS score, which ranges from 1 to 4 and represents the degree of urgency in patient attendance. Patients with PACS 1 are the most critically ill, those with PACS 2 are non-ambulant, those with PACS 3 are ambulant, and those with PACS 4 are non-emergencies. Our study focuses on patients presenting with chest pain, who routinely receive a 12-lead ECG investigation (Philips PageWriter TC50 Cardiograph) during triage and are placed in PACS 1 or 2 units where they receive further ECG monitoring (ZOLL X Series Monitor defibrillator). The study was approved by the local ethics committee (SingHealth Centralised Institutional Review Board, Singapore) with a waiver of patient consent.

### Patient recruitment and eligibility

All patients older than 21 years of age with a primary complaint of non-traumatic chest pain were eligible. Patients presenting in non-sinus rhythm (arrhythmias, asystole, complete heart blocks, or pacemaker rhythms) were excluded due to interference of these phenomena with the interpretation of QRS complexes. Similarly, patients with a high percentage of artifacts, ectopic beats, and non-sinus beats (>30 % of ECG recordings) were excluded due to their potential biasing effect on the HRV calculations [27]. Finally, patients who were lost to follow up or transferred to other (private) hospitals within the 30-day time frame were excluded, on account of inability to ascertain whether these patients had reached our primary endpoint.

### Data collection and processing

All data were collected on standardized forms in a Research Electronic Data Capture (REDCap) database. The electronic medical records (EMRs) were analyzed for demographic characteristics, medical history, presenting symptoms, clinical information, and laboratory results.

A trained research coordinator prospectively downloaded 12-lead ECG tracings from the ZOLL X Series monitor defibrillator on a daily basis. We use our in-house software package for ECG signal processing and parameter calculation [28]. Noise was manually removed from the lead II ECG tracing and its sample of 6 minutes was stored in an Excel (Microsoft Office 2007; Microsoft, Redmond, WA, USA) file for further processing. A 5–28 Hz band-pass filter was applied to the lead II sample to facilitate peak detection [29]. QRS complexes were detected using a threshold-plus-derivative method that has been previously validated [28]. Time domain and frequency domain HRV parameters were calculated in accordance with the guidelines outlined by the Taskforce of the European Society of Cardiology [30].

Vital signs were recorded at initial ED patient presentation using the Propaq CS Vital Signs Monitor (Welch Allyn, Skaneateles, NY, USA). The first set of complete vital signs obtained at initial presentation was used for this study. The 12-lead ECG tracings recorded during triage were used for the evaluation of ECG variables. These tracings were recorded using a Philips PageWriter TC50 cardiograph and subsequently extracted for analysis and storage. A trained research associate, blinded to patient outcomes, ascertained whether the patient was in sinus rhythm, and evaluated the 12-lead ECG tracings for abnormalities. We followed the definitions of ECG variables as described in John Hampton’s book “The ECG Made Easy”.

We tested the SEDRSM against the TIMI score for unstable angina (UA)/non-ST elevation myocardial infarction (NSTEMI), a score that has been employed on the ED to predict MACE within 30 days of presentation to the ED with chest pain [31]. Data pertaining to the TIMI score criteria were retrieved from the EMRs and used to construct the TIMI score.

### Outcomes

The primary endpoint of this study, MACE, was a composite outcome of death, acute myocardial infarction, and revascularization, including coronary artery bypass graft (CABG) or percutaneous coronary intervention (PCI), within 30 days of presentation to the ED. Patients were followed up and EMRs were reviewed to ascertain whether the patient had experienced an endpoint criterion within 30 days after presentation.

### Statistical analysis

SPSS (version 21.0; SPSS Inc, Chicago, IL, USA) software was used for statistical analysis. Derivation and validation of the SEDRSM was done in the same cohort. Univariate relationships between baseline characteristics and MACE were assessed using the appropriate statistical test, based on type and distribution of data. We tested normality of distribution by inspecting normality graphs and interpreting the Kolmogorov-Smirnov quantitative normality test.

A total of 16 HRV parameters, 13 ECG variables, 7 vital signs, and 3 demographic variables (age, gender, and race) were screened as candidate predictors of MACE using the same univariate analytical method described previously. Variables associated with a *p* value <0.05 were selected and categorized in order to facilitate scoring and increase applicability at the ED. HRV parameter category cutoffs were chosen based on the visual comparison between HRV parameter value and frequency of MACE occurrence. ECG variables were dichotomous. Vital signs and demographics were categorized based on recognized (physiological) cutoff values.

We introduced the categorized candidate variables into an automated likelihood ratio backward stepwise logistic regression model. The retained candidate variables were used to construct the SEDRSM. All unstandardized coefficients were normalized by dividing them by the smallest coefficient, and subsequently rounded off to the nearest integer. The SEDRSM score was then calculated by a simple arithmetic sum of the integers assigned to the criteria satisfied.

The overall goodness of fit of the model was assessed by the Hosmer-Lemeshow test. The predictive accuracy of the SEDRSM and TIMI score was assessed using the area under the receiver operating characteristic (AUROC) curve. Discriminatory values (i.e., sensitivity, specificity, positive predictive value, and negative predictive value) were also determined for both risk stratification models.

## Results

We included 763 patients in the study. The baseline characteristics of our total patient cohort and of those with and without a MACE (our primary endpoint) are shown in Table 1. A total of 254 patients experienced a MACE, versus a total of 509 who did not. The mean age of our cohort was 60 (SD = 13) years. The majority of the population was male (65 %). In the group that experienced a MACE, the patients were older (61.75 years) (*p* = 0.001), there were more male patients (72.8 %, *p* = 0.001) and more patients with diabetes mellitus (44.5 %, *p* = 0.001), and fewer patients with respiratory disease (1.6 %, *p* = 0.012). We also found that patients who experienced a MACE were more frequently admitted, specifically more often to general wards and intensive care wards. No other significant differences were found between the groups who did or not experience. The risk factors hypertension and hyperlipidemia were present in over half the cohort. The frequencies of all different types of MACE are shown in Table 2. The most frequent MACE was revascularization (24.5 %) by either PCI, or CABG, or both, followed closely by MI (23.6 %). Death (2.0 %) was the least frequent MACE experienced by patients in this cohort.

**Table 1:** Baseline characteristics of patients in the study

Characteristics	All patients	MACE	No MACE	*P* value
	(n = 763)	(n = 254)	(n = 509)	
Age in years, μ (σ)	60.49 (13.33)	61.75 (11.86)	59.86 (13.97)	0.001
Men	496 (64.9)	185 (72.8)	310 (60.9)	0.001
Race				0.647
Chinese	489 (64.1)	168 (66.1)	321 (63.1)	
Malay	144 (18.9)	47 (18.5)	97 (19.1)	
Indian	106 (13.9)	30 (11.8)	76 (14.9)	
Other	24 (3.1)	9 (3.5)	15 (2.9)	
Medical history				
IHD	336 (44.0)	115 (45.3)	221 (43.4)	0.643
DM	275 (36.0)	113 (44.5)	162 (31.8)	0.001
Hypertension	492 (64.5)	173 (68.1)	319 (62.7)	0.149
Hyperlipidemia	456 (59.8)	148 (58.3)	308 (60.5)	0.584
Previous stroke	60 (7.9)	19 (7.5)	41 (8.1)	0.887
Cancer	32 (4.2)	8 (3.1)	24 (4.7)	0.345
Respiratory disease	31 (4.1)	4 (1.6)	27 (5.3)	0.012
Renal disease	96 (12.6)	34 (13.4)	62 (12.2)	0.644
CHF	39 (5.1)	10 (3.9)	29 (5.7)	0.383
Previous PCI	175 (22.9)	64 (25.2)	111 (21.8)	0.315
Previous CABG	70 (9.2)	25 (9.8)	45 (8.8)	0.690
Previous MI	114 (14.9)	41 (16.1)	73 (14.3)	0.519
Disposition from ED				<0.001
Admission to GW	354 (46.4)	96 (37.8)	258 (50.7)	
Admission to ICW	176 (23.1)	141 (55.5)	35 (6.9)	
No admission	233 (30.5)	17 (6.7)	216 (42.4)	

Data are number (%) unless otherwise specified. Patients may have had more than one medical history and more than one disposition from the Emergency Department (ED). *P* values <0.05 were considered statistically significant. *MACE* major adverse cardiac event, *SD* standard deviation, *IQR* interquartile range, *IHD* ischemic heart disease, *DM* diabetes mellitus, *CHF* congestive heart failure, *PCI* percutaneous coronary intervention, *CABG* coronary artery bypass graft, *MI* myocardial infarct, *GW* general ward, *ICW* intensive care ward

**Table 2:** Frequency of MACE types within 30 days

Event	Number of patients (%)
Any MACE	254 (33.3)
Death	15 (2.0)
MI	180 (23.6)
PCI	161 (21.1)
CABG	29 (3.8)
Revascularization	187 (24.5)

Revascularization is a composite of percutaneous coronary intervention (PCI) and coronary artery bypass graft (CABG). Patients may have had more than one major adverse cardiac event (MACE). *MI* myocardial infarction

Table 3 shows the univariate association between vital signs, HRV parameters, 12-lead ECG variables, and our endpoint. A total of 19 candidates for the SEDRSM, including gender and age, were identified (*p* < 0.05). Heart rate and diastolic blood pressure were found to be significantly elevated in the cohort that experienced a MACE within 30 days. The presence of five ECG variables was strongly associated with the occurrence of a MACE within 30 days. Lastly, nine HRV parameters were found to be significantly different in the cohort that experienced a MACE versus the cohort that did not experience a MACE; these were average R-to-R interval (RR), SDRR (SD R-to-R interval), average HR, root mean square of successive differences (RMSSD), the number of interval differences of successive normal-to-normal (NN) intervals greater than 50 ms (NN50), the proportion derived by dividing NN50 by the total number of NN intervals (pNN50), triangular interpolation of NN interval histogram (TINN), very low frequency (VLF), and high frequency (HF).

**Table 3:** Comparison of vital signs, ECG variables, and HRV parameters in patients with and without MACE within 30 days of arrival at the ED

	No MACE	MACE	*P*-value
	(n = 509)	(n = 254)	
Vital signs, μ (σ)			
Temperature	36.4 (0.8)	36.4 (0.7)	0.517
Heart rate	75 (22)	81 (23)	0.001*
Respiratory rate	18 (1)	18 (1)	0.791
Systolic BP	139 (33)	138 (41)	0.690
Diastolic BP	75 (18)	78 (21)	0.005*
SpO2	99 (3)	99 (3)	0.505
Pain score	2 (4)	2 (5)	0.090
ECG variables, no. (%)			
ST elevation	13 (2.6)	52 (20.6)	<0.001*
ST depression	13 (2.6)	53 (20.9)	<0.001*
T inversion	82 (16.1)	69 (27.2)	<0.001*
Q wave	17 (3.3)	35 (13.8)	<0.001*
QT prolongation	159 (31.2)	102 (40.2)	0.015*
Left axis deviation	36 (7.1)	18 (7.1)	1.000
Right axis deviation	15 (2.9)	8 (3.1)	1.000
LBBB	3 (0.6)	2 (0.8)	1.000
RBBB	36 (7.1)	15 (5.9)	0.645
IVCD	3 (0.6)	11 (4.3)	0.001*
LAA	7 (1.4)	5 (2.0)	0.546
LVH	62 (12.2)	41 (16.1)	0.144
RVH	5 (1.0)	1 (0.4)	0.669
HRV parameters, μ (σ)			
Average RR	0.824 (0.241)	0.770 (0.233)	0.001*
SD RR	0.035 (0.028)	0.029 (0.031)	0.010*
Average HR	73.13 (21.16)	78.11 (23.31)	0.001*
SD HR	3.27 (2.66)	2.99 (2.30)	0.135
RMSSD	0.028 (0.031)	0.021 (0.031)	0.001*
NN50	10.0 (26)	6.0 (21)	0.018*
pNN50	2.85 (8.42)	1.55 (7.59)	0.013*
Triangular index	2.97 (1.96)	2.87 (1.94)	0.640
TINN	0.130 (0.118)	0.102 (0.113)	0.002*
Total power	0.470 (0.140)	0.485 (0.155)	0.428
VLF power	0.217 (0.169)	0.246 (0.202)	0.030*
LF power	0.113 (0.102)	0.110 (0.083)	0.265
Normalized LF	50.56 (30.52)	52.99 (35.85)	0.463
HF	0.113 (0.110)	0.098 (0.110)	0.029*
Normalized HF	49.44 (30.52)	47.01 (35.85)	0.463
LF/HF ratio	1.02 (1.33)	1.13 (1.68)	0.428

Vital signs and heart rate variability (HRV) parameters are expressed as mean (μ) and standard deviation (σ). *MACE* major adverse cardiac events, *BP* blood pressure, *ECG* electrocardiograph, *LBBB* left bundle branch block, *RBBB* right bundle branch block, *IVCD* intraventricular conduction defect, *LAA* left atrial abnormality, *LVH* left ventricular hypertrophy, *RVH* right ventricular hypertrophy, *HRV* heart rate variability, *NN* normal-to-normal, *SD* standard deviation, *RMSSD* root mean square of successive differences, *SpO*_*2*_ pulse arterial oxygen saturation, *TINN* triangular interpolation NN, *VLF* very low frequency, *LF* low frequency, *HF* high frequency. *P < 0.05

Ten of the original 19 candidate variables remained in the model after backwards variable elimination in the multivariable model. These were age, gender, heart rate, ST elevation, ST depression, Q wave, QT prolongation, average R-to-R interval (AVRR), Triangular interpolation NN (TINN), and high frequency (HF) (Table 4). The Hosmer-Lemeshow test indicated satisfactory fit (*p* = 0.282). Out of the ten predictors ST depression and ST elevation were the strongest with odds ratios of 10.83 (95 % CI 5.49, 21.36) and 8.48 (95 % CI 4.27, 16.85), respectively.

**Table 4:** Predictors of 30-day MACE after backwards elimination in multivariable regression

Variables	Unstandardized coefficients	*P* value	Adjusted OR (95 % CI)
Gender (male vs female)	0.773	<0.01	2.17 (1.45, 3.23)
Age, years (≥60 vs <60)	0.323	0.090	1.38 (0.95, 2.00)
Heart rate, beat/min		0.066	
≥115	0	-	1.00
≤55	1.510	0.038	4.52 (1.08, 18.89)
56–114	0.716	0.243	2.05 (0.62, 6.80)
ST elevation (yes vs no)	2.138	<0.001	8.48 (4.27, 16.85)
ST depression (yes vs no)	2.382	<0.001	10.83 (5.49, 21.36)
Q wave (yes vs no)	1.076	0.004	2.93 (1.41, 6.11)
QT prolongation (yes vs no)	0.372	0.054	1.45 (0.99, 2.12)
AVRR (<0.77 vs ≥0.77)	0.415	0.034	1.52 (1.03, 2.22)
TINN		0.006	
0.11–0.17	0	-	1.00
<0.11	0.712	0.002	2.04 (1.30, 3.20)
>0.17	0.277	0.279	1.32 (0.80, 2.18)
HF power (<0.07 vs ≥0.07)	0.531	0.007	1.70 (1.16, 2.50)

*MACE* major adverse cardiac events, *OR* odds ratio, *CI* confidence interval, *ED* emergency department, *AVRR* average RR, *TINN* triangular index NN, *HF* high frequency

The final set of criteria for the SEDRSM is shown in Table 5. The unstandardized coefficients were normalized by dividing the total set by 0.277, which was the smallest common multiplicative factor. The normalized unstandardized coefficients were subsequently rounded off to the nearest integer. The SEDRSM has a range of 0 to 37.

**Table 5:** Normalization of unstandardized coefficients and final corresponding SEDRSM scores

Model criteria	β Coefficients		Final score
Gender, male	0.773	2.789	3
Age in years, ≥60	0.323	1.165	1
Heart rate, beat/min			
≥115	0	0	0
≤55	1.510	5.448	5
56–114	0.716	2.582	3
ST elevation, yes	2.138	7.715	8
ST depression, yes	2.382	8.597	9
Q wave, yes	1.076	3.883	4
QT prolongation, yes	0.372	1.341	1
AVRR <0.77	0.415	1.498	1
TINN			
0.11–0.17	0	0	0
<0.11	0.712	2.571	3
>0.17	0.277	1.000	1
HF power <0.07	0.531	1.916	2

Risk score ranges from 0 to 37. *SEDRSM* Singapore emergency department risk stratification model, *AVRR* average RR, *TINN* triangular index NN, *HF* high frequency

**Fig. 1:**
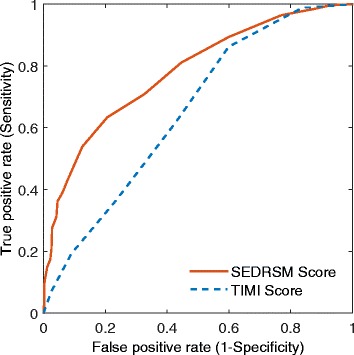
Receiver operating characteristic curves for the thrombolysis in myocardial infarct (*TIMI*) score and Singapore Emergency Department risk stratification model (*SEDRSM*) score

The SEDRSM performed with an AUROC (or *C* statistic) of 0.780 (95 % CI 0.743, 0.817), compared to an AUROC of 0.653 (95 % CI 0.611, 0.695) for the TIMI (UA/NSTEMI) score in the prediction of 30-day MACE. The SEDRSM performed significantly better than the TIMI score (*p* < 0.001). The ROC curves for the SEDRSM and the TIMI score are shown in Fig. 1.

**Fig. 2:**
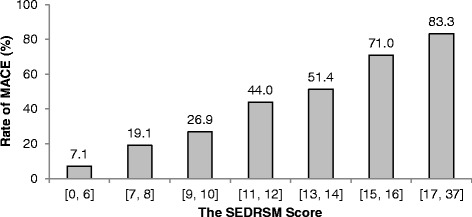
The relationship between the Singapore Emergency Department risk stratification model (*SEDRSM*) and the rate of major adverse cardiac events (*MACE*)

**Fig. 3:**
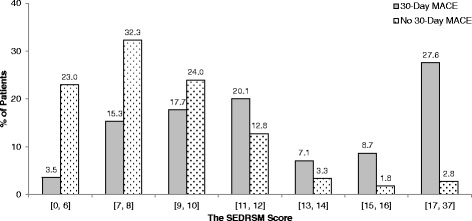
The distribution of the Singapore Emergency Department risk stratification model (*SEDRSM*) score in patients with and without major adverse cardiac events (*MACE*)

Figure 2 shows the SEDRSM score in relation to the rate of MACE. With an increase of the score constructed in the SEDRSM there is a proportionate increase in patients experiencing MACE. For example, 71.0 % of patients with the risk score of 15 or 16 have 30-day MACE. In Fig. 3, we did a further investigation on the performance of the SEDRSM where the distributions of the risk scores are illustrated by outcome categories, that is, with and without 30-day MACE. The gray bars indicate the score distributions for patients with MACE in terms of percentage of total number of patients with MACE falling in each bin of the SEDRSM score. The dotted bars indicate the score distributions for patients without MACE. As seen from Fig. 3, the percentage of patients without MACE decreases generally from 32.3 % to 1.8 % as the risk stratification score increases, whereas the percentage of patients with MACE fluctuates in a range from 3.5 % to 27.6 %. Due to small numbers of extreme scores we combined scores 0–6 and 17–37, and paired the remaining numbers for illustration.

Table 6 contains the discriminatory values of the SEDRSM and the TIMI score. The SEDRSM had better sensitivity of 0.709 (95 % CI 0.653, 0.765) and specificity of 0.674 (95 % CI 0.633, 0.715). The SEDRSM also had a higher positive predictive value of 0.520 (95 % CI 0.468, 0.573) and a negative predictive value of 0.823 (95 % CI 0.786, 0.859).

**Table 6:** Discriminatory values for the SEDRSM and TIMI scores

	SEDRSM	TIMI
AUROC (95 % CI)	0.780 (0.743, 0.817)	0.653 (0.611, 0.696)
Cutoff score	9	2
Sensitivity (95 % CI)	0.709 (0.653, 0.765)	0.618 (0.558, 0.678)
Specificity (95 % CI)	0.674 (0.633, 0.715)	0.572 (0.529, 0.615)
PPV (95 % CI)	0.520 (0.468, 0.573)	0.419 (0.369, 0.469)
NPV (95 % CI)	0.823 (0.786, 0.859)	0.750 (0.707, 0.793)

The Singapore Emergency Department risk stratification model (*SEDRSM*) has a range of 0 to 37; the thrombolysis in myocardial infarct (*TIMI*) score has a range of 0 to 7. *AUROC* area under the receiver operating characteristic, *CI* confidence interval, *PPV* positive predictive value, *NPV* negative predictive value

## Discussion

In this study we constructed a risk stratification model (the SEDRSM) incorporating vital signs, demographic data, ECG variables, and HRV parameters for the prediction of 30-day MACE in patients presenting to the ED with chest pain. In validation in the same cohort, the SEDRSM significantly outperformed the TIMI score in terms of AUROC (0.780 versus 0.653, *p* < 0.001). Additionally, the SEDRSM performs better than the TIMI score in terms of sensitivity, specificity, positive predictive value, and negative predictive value.

Vital signs and 12-lead ECG variables are well-established and frequently employed during clinical risk prediction on the ED [11, 32]. In contrast, many studies have reported the clinical and prognostic value of HRV parameters in the evaluation of patients with possible cardiovascular pathological conditions, but they have yet to be clinically applied [33]. This study demonstrates that HRV parameters can be successfully implemented in cardiovascular risk stratification on the ED, even amidst other established prognosticators. The TIMI score has been used as a benchmark due to its popularity and accuracy in predicting 30-day MACE in patients presenting to the ED with chest pain [31]. We used short-term (6-minute) recordings of 12-lead ECG as it has practical advantages in the time-scarce ED setting. Long-term (24-h) HRV analysis is prone to data analysis difficulties (e.g., failure to detect low-frequency oscillations and data-filtering difficulties). Recent evidence demonstrated a comparable predictive value for short-term vs long-term HRV analysis [34, 35]. The role of HRV in cardiovascular risk prediction has been examined previously but we cannot make direct comparisons with our study due to fundamental differences in methodology (i.e., no inclusion of 12-lead ECG variables) and a low yield of patients meeting the primary endpoints in previous reports [26].

Calculating HRV requires only ECG monitoring and standard analysis software [36, 37]. The SEDRSM had an AUROC of 0.780 versus an AUROC of 0.653 for the TIMI score. Risk stratification models are considered reasonable when the AUROC (or *C* statistic) is higher than 0.7 and strong when it exceeds 0.8 [38]. Our findings reaffirm the potential role of HRV amidst clinical cardiovascular predictors such as 12-lead ECG variables, and demonstrate a good performance compared to a widely used cardiovascular ED risk prediction model; however, there are still many opportunities for enhancement.

Our results show that three HRV parameters are significant predictors in a multivariable prediction model. Additionally, four 12-lead ECG variables, and two demographic variables were strong significant predictors in the multivariable model. Only one vital sign, heart rate, was incorporated as a criterion into the model. The heart rate can also be obtained through processing of the 12-lead ECG. This tool would allow wider diffusion of HRV parameters into clinical use as it enables HRV parameter interpretation by the physician, which is nearly impossible by manual methods. The SEDRSM allows early clinical anticipation of MACE on the ED, and thus facilitates early intervention.

HRV is a highly complex nonlinear phenomenon; even though time-domain and frequency-domain HRV parameters are most frequently employed (designated as the “classical analysis”) they are not fully representative due to their linear and stationary nature. Evidence suggests that nonlinear HRV parameters are much more suitable for the assessment and prognosis of cardiovascular risk than the “classical analysis” [39]. However, more high-quality longitudinal studies are required to establish the applicability of nonlinear HRV parameters [13].

Fragmented QRS complex is an easily obtainable ECG index that might further enhance cardiovascular risk prediction models [40]. Signal averaged ECG analysis has been demonstrated to have a high negative predictive value [41], and could be employed simultaneously with HRV processing. Microvolt T-wave alternans, a measure of repolarization dispersion, has been employed in the prediction of cardiac death and may add value to HRV and signal averaged ECG (SAECG) analysis [42]. Last, the occurrence of specific early repolarization morphological features that are frequently difficult to distinguish from true ST elevation, have recently been associated with increased risk of unexpected death. These patterns are considered particularly useful to refine risk stratification and identify a high-risk subset of patients; future research could explore the value of incorporating certain repolarization morphological features into risk prediction using the HRV and 12-lead ECG analysis [43, 44].

### Limitations

In our study we have compared the performance of the SEDRSM and the TIMI score for the prediction of 30-day MACE in patients presenting to the ED with chest pain. The SEDRSM score was only evaluated in our local population, whereas the TIMI score has been popular and widely validated, despite the fact that it was designed for a slightly different specification [4]. Though there is now evidence of its usefulness in this setting [31], the usefulness of some of the criteria in the TIMI score is questionable in the ED. For example, the TIMI score requires knowledge of results of prior cardiac catheterization, which might not be available. Newer risk stratification scores have been developed that are specifically calibrated towards patients presenting to the ED with chest pain. For example, the history, ECG, age, risk factors (HEART) score [45] and the Emergency Department assessment of chest pain score (EDACS) score [46] could serve as better benchmarks due to their applicability to, and specific design for, an ED setting in which the risk of 30-day MACE needs to be assessed [47].

Our study evaluates HRV as a quantitative measure of a supposed quantitative primary endpoint, MACE. However, MACE includes revascularization (either through PCI or CABG) as a category, which is not necessarily a purely quantitative endpoint. Patient choice, i.e., the decision by patients to reject revascularization at their own risk, is a qualitative phenomenon. The revascularization endpoint therefore interferes with the computation of quantitative linear relationships between predictor and endpoint. In our study we have not corrected for, or quantified the number of patients rejecting medical diagnostics or therapy at any point after presentation to the ED. A modification of endpoint or exclusion of these patients would likely facilitate a more accurate linear relationship between predictor and endpoint.

In addition to the above limitations, we note that HRV parameters can not be manually calculated or interpreted. In this study, we used our in-house software to derive HRV measurements. We are currently developing a portable hardware device to integrate data acquisition and analysis. We believe that such a device will help clinicians quickly identify patients at high risk of developing MACE.

## Conclusions

In our single-center, single-cohort study of patients presenting to the ED with chest pain, our risk stratification model (the SEDRSM) outperformed the TIMI score for the prediction of MACE, a composite endpoint of MI, revascularization, and death (AUROC of 0.780 versus 0.653 respectively). The SEDRSM incorporated the following criteria: age, gender, heart rate, three HRV parameters, and four 12-lead ECG variables. The SEDRSM provides useful information for making decisions about the placement of ED patients with chest pain under observation, and in determining the therapeutic strategy. The SEDRSM contains eight criteria that can be acquired by processing electrocardiographic data, allowing for a 12-lead ECG-based risk prediction device, setting aside only the manual input of demographic criteria. A risk stratification device could employ machine learning techniques that reduce information loss occurring during the construction of multivariable linear association models. There are several other ECG-based variables that can be valuable additions to our risk stratification score; these include nonlinear HRV parameters and novel depolarization or repolarization ECG variables. Our study demonstrates the potency and suitability of the SEDRSM for cardiovascular risk prediction in the ED, but also warrants evaluation and possibly resolution of existing limitations before it can be implemented in clinical practice.

## Key messages

- The Singapore General Hospital (SGH) Emergency Department risk stratification model (SEDRSM) was proposed for patients with chest pain
- The SEDRSM outperformed the TIMI score in predicting 30-day major adverse cardiac events

## Abbreviations

ACC/AHA, American College of Cardiology/American Heart Association; BP, blood pressure; CABG, coronary artery bypass graft; CHF, congestive heart failure; CI, confidence interval; DM, diabetes mellitus; ECG, electrocardiogram; ED, emergency department; EDACS, Emergency Department assessment of chest pain score; EMR, electronic medical records; GRACE, global registry of acute coronary events; GW, general ward; HEART, history, ECG, age, risk factors, Troponin; HF, high frequency; HR, heart rate; HRV, heart rate variability; ICW, intensive care ward; IHD, ischemic heart disease; IVCD, intraventricular conduction defect; LAA, left atrial abnormality; LBBB, left bundle branch block; LF, low frequency; LVH, left ventricular hypertrophy; MACE, major adverse cardiac event; MI, myocardial infarction; NHCS, National Heart Centre Singapore; NN, normal-to-normal intervals; NN50, number of interval differences of successive NN intervals greater than 50 ms; PACS, patient acuity category scale; PCI, percutaneous coronary intervention; pNN50, proportion derived by dividing NN50 by the total number of NN intervals; PURSUIT, Platelet glycoprotein IIb/IIIa in unstable angina: receptor suppression using integrilin therapy; RBBB, right bundle branch block; REDCap, research electronic data capture; RMSSD, root mean square of successive differences; ROC, receiver operator characteristic; RR, R-to-R interval; RVH, right ventricular hypertrophy; SAECG, signal averaged ECG; SDRR, standard deviation R-to-R interval; SEDRSM, Singapore Emergency Department risk stratification model; SGH, Singapore General Hospital; TIMI, thrombolysis in myocardial infarct; TINN, triangular interpolation of NN interval histogram; UA/NSTEMI, unstable angina/non-ST-elevation myocardial infarct; VLF, very low frequency
